# Emergence of a novel reassortant H3N3 avian influenza virus with enhanced pathogenicity and transmissibility in chickens in China

**DOI:** 10.1186/s13567-025-01484-1

**Published:** 2025-03-11

**Authors:** Chunping Zhang, Conghui Zhao, Jiacheng Huang, Yang Wang, Bo Jiang, Hangyu Zheng, Mingzhi Zhuang, Yanni Peng, Xiaoxuan Zhang, Sha Liu, Haoxi Qiang, Huanhuan Wang, Xiancheng Zeng, Guijie Guo, Ji-Long Chen, Shujie Ma

**Affiliations:** Fujian Province Joint Laboratory of Animal Pathogen Prevention and Control of the “Belt and Road”, College of Animal Sciences, Fujian Agriculture and Forestry University, Fuzhou, 350002 Fujian China

**Keywords:** Zoonoses, avian influenza virus, H3N3, evolution, pathogenicity, transmissibility

## Abstract

H3N3 avian influenza viruses (AIVs) are less prevalent in poultry than H3N8 viruses. However, although relatively rare, reassortant H3N3 viruses have been known to appear in both domestic poultry and wild birds. In this study, we isolated the H3N3 virus in chickens sourced from a live poultry market in China. A comprehensive genomic analysis revealed that the virus possessed a single basic amino acid in the cleavage site of the hemagglutinin (HA) gene. Phylogenetic analysis indicated that eight genes in the H3N3 virus belong to the Eurasian lineage. Specifically, the HA and NA genes were clustered with H3N2 and H11N3, respectively, while the internal genes were closely related to the H3N8 and H9N2 viruses. Furthermore, the H3N3 virus exhibited high and moderate stability in thermal and acidic conditions and efficient replication capabilities in mammalian cells. The H3N3 virus demonstrated that it could infect and replicate in the upper and lower respiratory tract of BALB/c mice without prior adaptation, triggering hemagglutination inhibition (HI) antibody titres ranging from 80 to 160; notably, the H3N3 virus replicated vigorously within the chicken respiratory and digestive tracts. The virus also transmitted efficiently and swiftly among chickens through direct contact, leading to higher levels of HI antibodies in both the inoculated and contact birds. These findings suggest that the H3N3 virus may be a novel reassortant originating from viruses circulating in domestic poultry, thus demonstrating an increased pathogenicity and transmissibility in chickens. Our study determines that H3N3 AIV potentially threatens the poultry industry and public health, highlighting the importance of active surveillance of AIVs.

**Supplementary Information:**

The online version contains supplementary material available at 10.1186/s13567-025-01484-1.

## Introduction

Avian influenza virus (AIV) belongs to the *Orthomyxoviridae* family and has a genome of eight negative-sense segmented RNAs. The viral genome primarily encodes the polymerase basic protein 2 (PB2), polymerase basic protein 1 (PB1), polymerase acidic protein (PA), hemagglutinin (HA), nucleoprotein (NP), neuraminidase (NA), matrix protein 1 (M1), matrix protein 2 (M2), non-structural protein 1 (NS1), and non-structural protein 2 (NS2). Currently, 16 distinct HA and 9 NA subtypes of influenza viruses have been detected in avian species. Among these subtypes, virus strains of H5 and H7 subtypes are classified as highly pathogenic avian influenza viruses (HPAIV) [1–3]. Moreover, the H17N10 and H18N11 subtypes have been exclusively identified in bats, demonstrating the extensive diversity of the influenza virus across different species [4, 5]. Another example of this diversity is the H19 subtype virus detected in cloacal swabs of wild birds based on nucleotide sequences [6, 7]. Furthermore, the simultaneous infection of a single cell by multiple viruses, particularly strains from different subtypes, enhances the likelihood of genetic reassortment, thereby promoting the genesis of novel strains of AIVs [8]. The recent emergence of reassortant viruses, including H3N8, H5N1, H5N6, H5N8, and H7N9, has significantly damaged the animal breeding industry and posed substantial threats to public health [1, 2, 9–15].

The H3 subtype of AIVs has been persistently identified in both wild birds and domestic poultry across China, with the H3N2 and H3N8 strains emerging as the most predominant subtypes [10, 16–19]. In recent years, there have been cases of human infection with the H3N8 subtype of AIVs. A 56-year-old woman who had undergone immunosuppression died due to the virus, underscoring the critical role of the H3 subtype virus in interspecies transmission and its potential risks for public health [15, 20–25]. The first H3N3 virus with a confirmed sequence of HA was A/duck/Hong Kong/22A/1976 (H3N3) in the GenBank database (GenBank accession number: AB292410.1). Since this confirmed sequence, H3N3 AIVs have been widely prevalent across a range of hosts, including domestic poultry [26–28], wild birds [29], seals [30], pigs [31], and humans (accession number from GenBank: MK648248.1, MH685471.1; accession number from Global Initiative on Sharing All Influenza Data (GISAID): EPI904032, EPI351634).

Several H3N3 AIVs have been recently detected in China. Mao and colleagues reported that a novel reassortant H3N3 AIV demonstrated increased pathogenicity in chickens, with the HA and NA genes being genetically similar to the human infected isolates H3N8 and H10N3, while the internal genes (PB2, PB1, PA, NP, M, and NS) were derived from H9N2 circulating in chickens in China [26]. Li and colleagues identified an H3N3 virus in environmental samples taken in Hebei. The virus comprised the HA and NA genes, provided by H3N8 and H10N3, respectively, with its genes encoding internal proteins from genotype G57 of H9N2 [27]. In this study, we detected and isolated an H3N3 virus in chickens from a live poultry market in China's Fujian province.

To investigate the biological traits of the H3N3 virus, we performed experiments including phylogenetic analysis, pathogenicity in cells and mice, viral stability, and transmission in chickens to evaluate the potential threat of the H3N3 virus to the poultry industry and public health. Our findings indicate that the novel H3N3 virus exhibits enhanced pathogenicity and transmissibility in chickens. These findings will help us better understand the properties of the H3N3 virus and highlight the need for continuous surveillance of AIVs circulating in domestic birds.

## Materials and methods

### Cells, eggs, animals, and facilities

Madin-Darby canine kidney (MDCK) and human lung adenocarcinoma epithelial (A549) cells were grown in Dulbecco’s modified Eagle’s medium (DMEM) supplemented with 10% foetal bovine serum and antibiotics at 37 ℃ with 5% CO_2_. Specific pathogen-free (SPF) embryonated chicken eggs were obtained from Jinan SPAFAS Poultry Co., Ltd. Six-week-old female BALB/c mice were purchased from Zolgene Biotechnology Co., Ltd, Fuzhou, China. The rabbit anti-NP polyclonal antibody was produced and stored in our laboratory, as described previously [32]. The biosafety-level independent air-supply isolation mouse cage (375 mm × 160 mm × 180 mm) and GC-type individually ventilated cages (IVC) mouse cage (bottle outside) used in our study were purchased from Suzhou Fengshi Laboratory Equipment Co., Ltd. The chicken sterile isolator (1500 mm × 860 mm × 1500 mm) was purchased from Suzhou Suhang Technology Equipment Co., Ltd. All conditions for animal housing and experimental operations comply with ethical standards for animal welfare.

### Sample collection and virus isolation

The samples were collected from chickens at a live poultry market in Fujian province, China. Each sample was placed in 2 mL of phosphate-buffered saline (PBS) supplemented with penicillin (2000 U/mL) and streptomycin (2000 U/mL). The samples were maintained at 2–8 ℃ and transported to the laboratory in sealed containers. All samples were centrifuged at 4 ℃ at 3000 × *g* for 10 min. Then, 200 μL of the supernatants were inoculated into 10-day-old embryonated chicken eggs for 48 h at 37 ℃. The allantoic fluid was collected and tested for HA activity with 1% chicken red blood cells (RBCs). Virus stocks were propagated in 10-day-old SPF embryonated chicken eggs and stored at −70 ℃ before use. The samples and live virus experiments were conducted within the enhanced biosafety level 2 (BSL2 +) laboratory at Fujian Agriculture and Forestry University.

### Sequencing and phylogenetic analysis of the H3N3 virus

Viral RNAs were extracted using the TIANamp Virus RNA Kit (TIANGEN, Beijing, China), and standard reverse transcription polymerase chain reaction (RT-PCR) was performed with primers for AIVs (primer sequences available on request). Eight influenza virus gene segments were sequenced in Sangon Biotech Co., Ltd. The nucleotide sequences were assembled using the SEQMAN program (DNASTAR, Madison, WI, USA). Phylogenetic trees were constructed using MEGA software (Version 11) with the Neighbor-Joining method. A bootstrap value of 1000 was used to estimate the statistical reliability of clades, and values higher than 90 were automatically shown above or below the branch. The trees of HA, NA, and internal genes were rooted to A/equine/Uruguay/1/1963(H3N8), A/tern/South Africa/1959(H5N3), and A/equine/Prague/1956(H7N7), respectively. The sequences of the H3N3 virus were deposited in GISAID databases under accession numbers EPI3523562–EPI3523569.

### Viral thermostability assay

The H3N3 virus stock was standardised to 64 HA units (HAU) by diluting in PBS to assess viral thermostability. The virus was then incubated at 56 °C in a water bath for various intervals (0, 5, 10, 30, 60, 90, 120, 150, 180, and 240 min). Subsequently, 25 μL of the heat-treated virus was transferred to 96-well plates and serially diluted two-fold in 25 μL of PBS, with each sample in triplicate. After dilution, 25 μL PBS was added to each well. The hemagglutination activity was tested by adding 25 μL of 1% RBCs to each well, followed by incubation between 15 to 30 min at room temperature. We determined the viral infectivity after heat treatment at 56 °C by conducting titrations in MDCK cells, and each sample was tested three times. We conducted three independent experiments to reduce the likelihood of potential biases.

### Viral acid stability assay

The virus stock was diluted using PBS to 6 log_10_ 50% egg infectious doses (EID_50_). The pH levels of the diluted viral stocks were carefully adjusted to the specified values (4.8, 5.0, 5.2, 5.4, 5.6, 5.8, 6.0, 6.2, 6.4, 6.6, and 7.2) through the gradual, dropwise addition of 0.1 M citric acid. Then, the viruses with indicated pH levels were incubated at 37 °C for 1 h. After the acid treatment, 100 μL of the virus-containing sample was transferred to 900 μL DMEM to re-neutralise the sample as previously described [33, 34]. Viral infectivity was determined by titration in MDCK cells, with each sample in triplicate. Three independent experiments were performed to minimise potential biases. As a negative control, the PBS was adjusted to indicated pH values and re-neutralised with DMEM as described above. The tenfold diluted re-neutralised PBS was subjected to testing cell viability.

### Cell viability assay

Cell viability was measured using Cell Counting Kit-8 (Dojindo, Shanghai, China) according to the manufacturer’s protocol. MDCK cells were seeded on a 96-well plate and treated with PBS that had been re-neutralised, as prepared in the viral acid stability assay, for 1.5 h. Washed thrice with PBS, the 96-well plate was cultured in a 37 °C, 5% CO_2_ incubator for 24 h. Subsequently, a highly water-soluble tetrazolium salt (WST-8) solution was added to each well, and the cells were incubated at 37 °C for 1 h. Absorbance was measured using the Tecan Infinite M200 Pro system.

### Virus replication curves in cells

MDCK and A549 cells were grown on 12-well plates and infected with the virus at a multiplicity of infection (MOI) of 0.01. The inoculum was removed after 1 h of incubation. After three washes with PBS, the cells were supplemented with DMEM containing 0.125 μg/mL tosyl sulfonyl phenylalanyl chloromethyl ketone (TPCK) treated trypsin (Sigma, St. Louis, MO, USA) and were incubated at 37 ℃. Virus-containing cell culture supernatant was collected at 12 h, 24 h, 36 h, and 48 h post-inoculation (hpi) and titrated in MDCK cells. The growth curves represent the combined results from three independent experiments.

### HA and HA inhibition (HI) assay

For the HA assay, 50 μL PBS was added to wells 2–11 of each row in a 96-well V-bottomed microtiter plate, with 100 μL of the indicated virus added to the first well. Then, serial two-fold dilutions were made by transferring 50 μL from the first well of each row to their successive wells. The final 50 μL mixture was discarded. As a negative control, no virus was added to row 12. We then added 50 μL 0.5% RBCs to each well and mixed the wells by manually agitating the plates. The plates were then incubated at room temperature for 15 min. The highest dilution induced by the agglutination of the RBCs was the HA titre of the virus.

For the HI assay, 25 µL of PBS was added to wells 2–11 of every row in a 96-well V-bottomed microtiter plate. Next, 50 µL of the indicated serum was added to the first well. Serial two-fold dilutions of the treated sera were prepared by transferring 25 µL from the first well to the successive wells of each column. The final 25 μL mixture was discarded. Instead of treated sera being added to the final well of the plate, 25 µL PBS was added to be used as serum controls. Next, we added 25 µL of a four-unit standardised antigen to all the wells in the plates. The contents were then mixed by manually agitating the plates. Plate incubation was at room temperature for 30 min. Then, 25 µL 0.5% RBCs was added to all wells and incubated at room temperature for 15 min. The highest dilution preventing the RBCs’ agglutination is the serum’s HI titre.

### Mouse study

Six-week-old female BALB/c mice were inoculated intranasally with 10^6^ EID_50_ of the virus in a volume of 50 μL. Briefly, three mice were euthanised at 3 days post-inoculation (dpi), and the brain, nasal turbinate, lung, spleen, and kidney were collected for virus titration in embryonated chicken eggs. Mouse lungs collected at 3 dpi were fixed in 10% formalin and then stained with haematoxylin and eosin (H&E) and immunohistochemistry (IHC) for histological analysis. The remaining five mice were monitored daily for 14 days regarding weight loss and survival. Mice inoculated with PBS were used as a control group to observe body weight and pathological changes. Sera were collected at 21 dpi for HI antibody detection. Mice sera were treated with receptor-destroying enzyme (RDE) (Denka-Seiken, Japan) according to the WHO protocol to remove the non-specific agglutinins before performing HI assays.

### Chicken study

Three 3-week-old SPF chickens were intranasally inoculated with 10^6^ EID_50_ of the virus in a volume of 200 μL to investigate the replication and pathogenicity of the virus in chickens. The organs, including the brain, trachea, lung, spleen, liver, pancreas, kidneys, intestine, rectum, and bursa of Fabricius, were collected for virus titration in eggs at 3 dpi. For the transmission study, three chickens were inoculated with 10^6^ EID_50_ of the virus in a volume of 200 μL. Three naive chickens of the contact group were placed together in an isolator at 24 hpi. Oropharyngeal and cloacal swabs were taken from the inoculated and contact group of chickens at 2-day intervals, beginning on 2 dpi (1 day post-exposure). The viral titres of the swabs were titrated in embryonated chicken eggs. Sera were collected on 10, 15, and 21 dpi, and the antibody titres were determined using the HI test.

## Results

### Global prevalence of H3 subtype viruses

**Figure 1:**
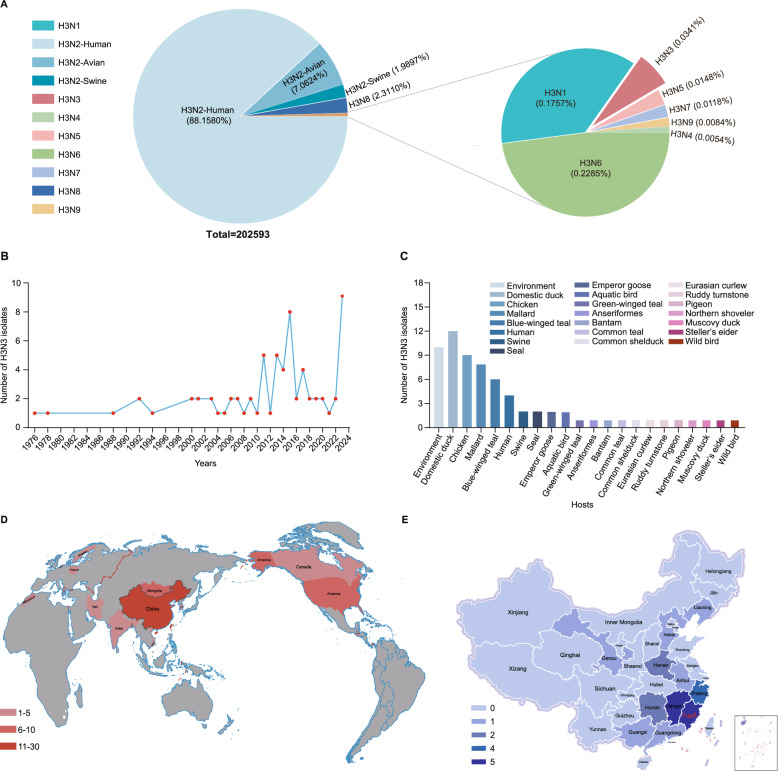
**Global prevalence of H3 subtype viruses**. **A** The HA and NA combinations of H3Ny strains in the GISAID and GenBank database. **B** The isolation years of the H3N3 viruses. **C** The hosts of H3N3 viruses. **D** The global distribution of the H3N3 subtype viruses. **E** The geographic distribution of the H3N3 subtype viruses in China. Sequences of the H3 subtype viruses were retrieved in GISAID and GenBank databases to analyse the viruses’ host, distribution, and isolation date. All the public data utilised in this study from GISAID and GenBank were current as of August 31, 2024.

To comprehensively understand the global prevalence of H3 subtype AIVs, all available sequences of H3 AIVs were retrieved from the GISAID and GenBank databases, covering the period from 1976 to 2024 for further analysis. The results indicated that over 97% of the H3 viruses were of the H3N2 subtype, including seasonal H3N2 viruses (88.1580%), H3N2 AIVs (7.0624%), and H3N2 viruses detected in pigs (1.9897%) (Figure 1A). Furthermore, the proportion of H3N3 subtype AIVs is relatively low, accounting for only 0.0341% of all H3 subtype viruses. The H3N3 virus has predominantly been identified within the last decade, signifying its emergence as a notable subtype in recent years (Figure 1B). Our analysis of the host spectrum indicated that wild birds and domestic poultry are the main hosts for H3N3 viruses. Notably, only eight strains of H3N3 viruses were detected in mammals, including pigs, seals, and humans (Figure 1C). Furthermore, geographical distribution results indicated that the H3N3 subtype viruses were predominantly found in North America and Asia (Figure 1D). In China, the H3N3 viruses, including the H3N3 isolate in this study, were primarily detected in southeastern China (Figure 1E).

### Isolation and phylogenetic analysis of the H3N3 virus

In March 2023, we detected and identified an H3N3 virus in a sample collected from the cloacal chicken at a live poultry market in Fujian province, China. Positive samples were amplified in SPF embryonated chicken eggs to prepare the virus stock, and the HA and NA subtypes were identified through Sanger sequencing. The virus was designated as A/chicken/Fujian/C80/2023 (H3N3) (CK/FJ/C80/2023). To systematically elucidate the evolution of the H3N3 virus, we conducted a phylogenetic analysis of the eight genes.

**Figure 2:**
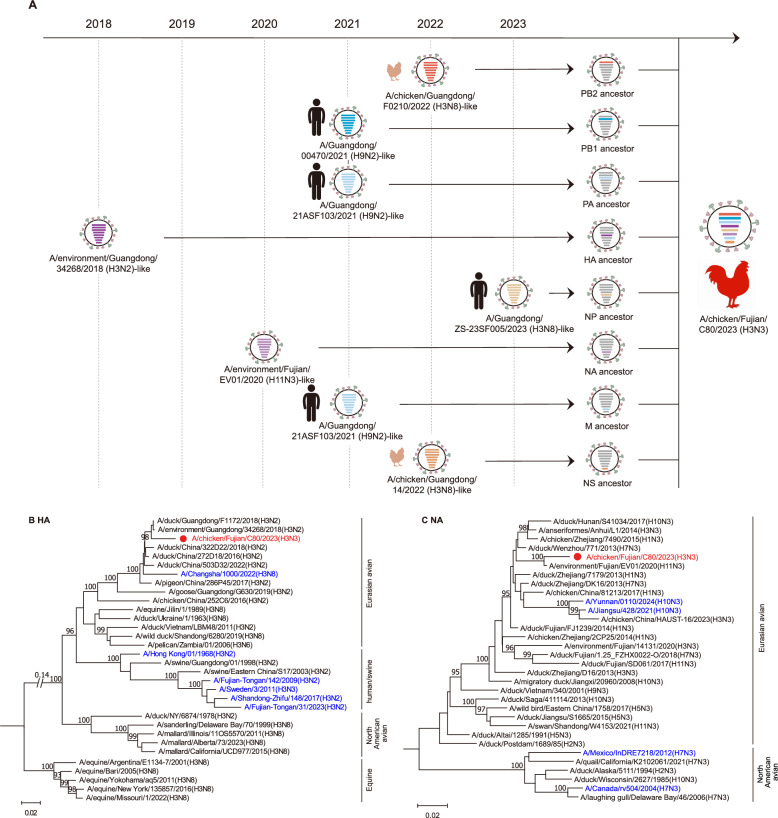
**Phylogenetic analysis of HA and NA genes of H3N3 virus**. **A** A simplified schematic showing the putative genomic composition of the H3N3 virus. The bars represent eight gene segments of AIVs (from top to bottom: PB2, PB1, PA, HA, NP, NA, M, and NS), and the bar’s colour indicates the gene segment’s closest donor strain. Phylogenetic trees were constructed using MEGA software (Version 11) with the Neighbor-Joining method. A bootstrap value of 1000 was used to estimate the statistical reliability of clades, and values higher than 90 were shown above the branch. The tree of **B** HA and **C** NA were rooted to A/equine/Uruguay/1/1963(H3N8) and A/tern/South Africa/1959 (H5N3), respectively. The H3N3 virus isolated in this study is shown in red, and viruses shown in blue are human isolates. The regions of nucleotide sequence used for the phylogenetic analyses were HA, 29–1732 and NA, 19–1431.

A basic local alignment search tool (BLAST) search conducted on the GISAID and GenBank databases revealed a notable degree of nucleotide sequence similarity between the CK/FJ/C80/2023 strain and a range of subtype viruses, including H3N2, H11N3, H3N8, and H9N2. These viruses were identified in environmental samples, chickens, and humans across China (Figure 2A and Additional file 1). Furthermore, the HA and NA genes showed relatively high similarity to H3N2 (97.18%) and H11N3 (98.16%) viruses isolated in environmental samples in Guangdong and Fujian, China, respectively.

**Figure 3:**
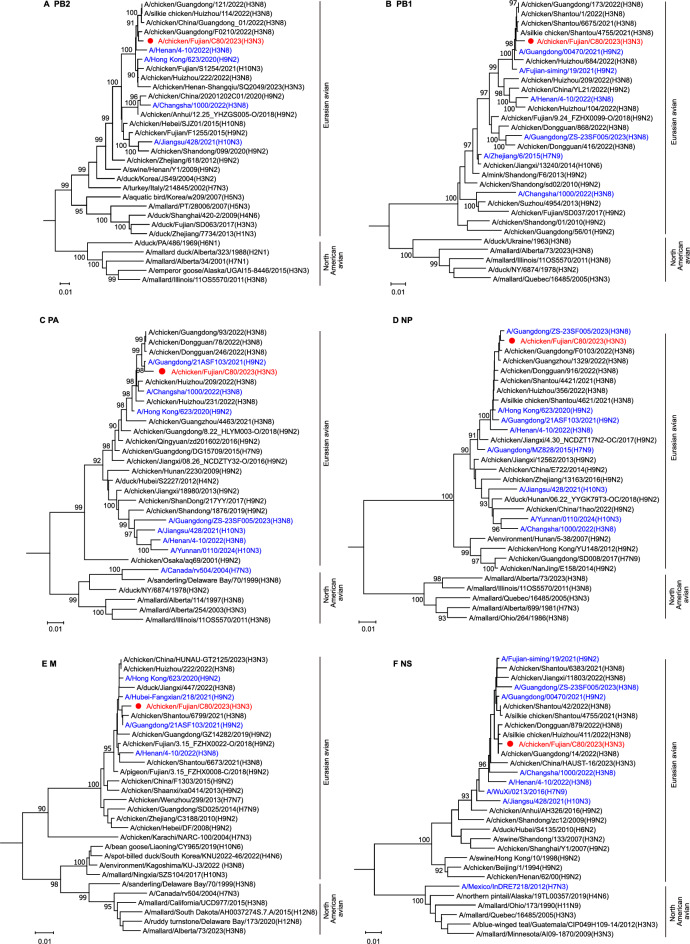
**Phylogenetic analysis of the internal genes of the H3N3 virus**. Phylogenetic trees were constructed using MEGA software (Version 11) with the Neighbor-Joining method. Bootstrap value of 1000 was used to estimate the statistical reliability of clades, and values higher than 90 were automatically shown above or below the branch. The regions of nucleotide sequence used for the phylogenetic analyses were as follows: PB2, 37–2307; PB1, 25–2298; PA, 25–2175; NP, 46–1542; M, 26–1007; and NS, 29–849. The trees of **A** PB2, **B** PB1, **C** PA, **D** NP, **E** M, and **F** NS were rooted to A/equine/Prague/1/1956 (H7N7). The virus in red is used in this study, and viruses in blue are human isolates.

In contrast, the internal genes exhibited greater similarity to the H3N8 and H9N2 viruses (99.12%−99.76%) (Figure 2A and Additional file 1). Notably, all eight genes of the CK/FJ/C80/2023 virus belong to the Eurasian lineage and are markedly distinct from the North American lineage (Figures 2 and 3). Therefore, we constructed phylogenetic trees using the Neighbour-Joining method to understand better the genesis and evolutionary characteristics of the H3N3 virus. Our findings indicated that the HA gene of the H3N3 virus was most closely related to the isolate of H3N2-like viruses, which clustered with the H3N8 virus detected in humans in Changsha (Figure 2B). The NA gene was closely related to the H11N3 virus identified in environmental samples, and it grouped with the H10N3 viruses detected in human cases (Figure 2C). The PB2 and NS genes were most similar to H3N8 viruses isolated from chickens (Figures 3A and F). Conversely, the NP gene showed the closest relationship with the H3N8 virus found in humans (Figure 3D). Furthermore, the PB1, PA, and M genes exhibited the greatest affinity to H9N2 viruses detected in human infections (Figures 3B, C and E).

These results imply that the CK/FJ/C80/2023 virus is a novel strain that has emerged through complex reassortment involving viruses from multiple origins. Moreover, the phylogenetic analysis suggests the virus may share common ancestors with the H3N8 and H9N2 human isolates, thus highlighting its zoonotic potential.

### Molecular characteristics of the H3N3 virus

**Figure 4:**
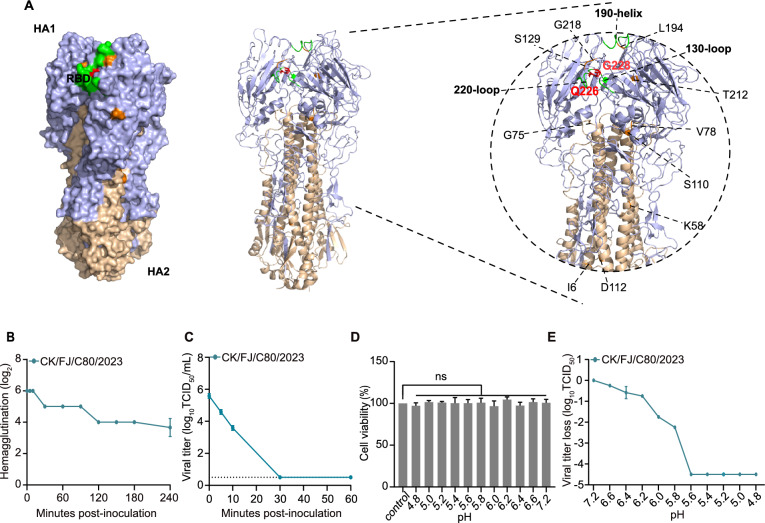
**Homology modelling structural analysis of HA and stability of the H3N3 virus**. **A** The three-dimensional structure of the HA protein of the H3N3 virus was predicted using Swiss-model homology modelling (PDB: 4wa1). The HA protein’s three-dimensional structure diagram was drawn using PyMOL (Version 2.5.7). Colours indicate the specific locations of the receptor binding site in red and key sites influencing viral stability on the HA protein surface. H3N3 virus was standardised to 64 HA units by diluting stock virus in PBS and then incubated at 56 °C in a water bath for indicated times. The HA titre was tested by adding 25 μL of 1% RBCs to each well, followed by incubation for 15 to 30 min at room temperature. Viral infectivity after heat treatment at 56 °C was determined by titration in MDCK cells in triplicate. **B** Viral HA titres at the indicated time following treatment at 56 °C in a water bath. **C** Viral titres at the indicated time were determined in MDCK cells after treatment at 56 °C in a water bath. **D** Cell viability of negative control of PBS re-neutralised by DMEM. MDCK cells were seeded on a 96-well plate and treated with re-neutralised tenfold diluted PBS prepared in the viral acid stability assay for 1.5 h. After three washes with PBS, the 96-well plate was cultured in a 37 °C, 5% CO2 incubator for 24 h. Subsequently, cell viability was measured using Cell Counting Kit-8 according to the manufacturer’s protocol. **E** Virus titre loss at the indicated pH value was determined in MDCK cells. The diluted viral samples were adjusted to indicated values by gradual, dropwise addition of 0.1 M citric acid. Then, the virus was incubated at 37 °C for 1 h. Viral infectivity was determined by triplicate titration in MDCK cells. Three independent experiments were conducted to minimise potential biases.

The amino acid residues found at the cleavage site of the HA protein are PEKQTR↓GLF, indicating low pathogenicity in chickens (Additional file 2). The key amino acids at the receptor binding site in the HA protein are Q226 and G228 (following H3 numbering), suggesting that the H3N3 may preferentially bind to the avian-type receptor (α−2,3 sialic acid) (Figure 4A and Additional file 2). In this study, the key amino acids influencing HA stability were screened. Specifically, the mutations L194 and T212 in the HA1 sub-unit and I6 and G75 in the HA2 sub-unit were identified as key determinants for HA stability. Our findings suggest that the CK/FJ/C80/2023 virus exhibits amino acid characteristics that maintain viral thermal and acid stability. Furthermore, various substitutions for mammalian adaptation were found in PB2, PB1, PA, NP, NA, M1, M2, and NS1 (Additional file 2). The potential glycosylation site motifs N-X-S/T were found to occur at seven sites in HA protein at positions 24, 38, 54, 181, 301, and 499, and at five sites in the NA protein at positions 57, 66, 72, 146, and 308 (Additional file 3).

### Viral stability of the H3N3 virus

Numerous studies have revealed that HA stability is a crucial determinant in virus host range, infectivity, and transmissibility [35–37]. To assess the potential risks of the H3N3 virus, we conducted assays to evaluate its thermostability and acid stability. The H3N3 virus was standardised to 64 HAU in PBS and incubated at 56 °C in a water bath for the previously indicated time intervals. According to previous studies, AIVs can be categorised into three types based on whether two titres can reduce the HA titre after incubating in a water bath for 5 and 30 min at 56 °C [35, 37]. The first category comprises thermally unstable viruses, which show a decrease in HA titre at two different titres within 5 min at 56 °C. The second category includes thermally stable viruses, characterised by a two-titre reduction in the HA titre after 30 min at 56 °C. The third category virus was classified as moderately thermally stable, displaying intermediate characteristics between the two types. The HA titre remained at 64 HAU after a 10 min incubation at 56 °C and dropped to 32 HAU after 30 min incubation (Figure 4B). This outcome indicates that the virus shows thermostability as the HA titre decreased only once in 30 min at 56 °C.

Viral infectivity was also evaluated to confirm the viral stability further. The findings demonstrated that the H3N3 virus maintained its infectivity following a 10-min incubation at 56 °C, yet it lost infectivity after 30 min incubation at the same temperature (Figure 4C). We used PBS as the negative control for testing cell viability. The results indicated that the re-neutralised PBS had no significant effects on cell viability (Figure 4D). The acid stability result showed that the H3N3 virus began to lose infectivity in the pH 6.6 buffer. The viral infectivity consistently decreased with the reduction in pH and was entirely lost when the pH fell to 5.6 (Figure 4E), indicating that the H3N3 virus demonstrated moderate acid stability.

These findings highlight that the newly identified reassortant H3N3 virus possesses a high degree of thermostability.

### Replication of the H3N3 virus in mammalian cells

**Figure 5:**
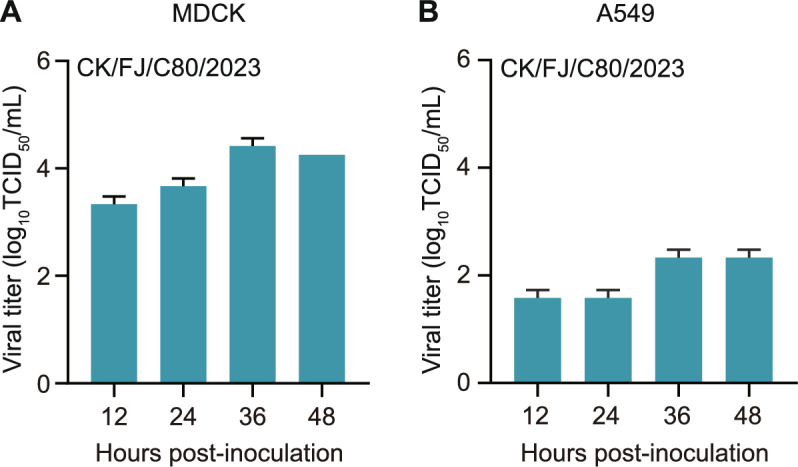
**Replication of the H3N3 virus in mammalian cells**. **A** MDCK and **B** A549 cells were infected with the H3N3 virus at an MOI of 0.01 in triplicate. Then, the supernatants from the infected MDCK and A549 cells were collected at the indicated times for titration in MDCK cells.

Multicycle growth curves of the CK/FJ/C80/2023 virus were evaluated in MDCK and A549 cells to test the replicative kinetics of the H3N3 virus in mammalian cells. Briefly, the cells were grown on 12-well plates and infected with the virus at an MOI of 0.01. The supernatants were collected at indicated times and titrated in MDCK cells. The titres of CK/FJ/C80/2023 virus in MDCK and A549 cells ranged from 3.25 to 4.50 log_10_ TCID_50_/mL (Figure 5A) and from 1.50 to 2.50 log_10_ TCID_50_/mL (Figure 5B) at 12 h, 24 h, 36 h, and 48 hpi, respectively. The virus titres in MDCK cells were relatively higher than in the A549 cells. These results indicated that the H3N3 virus could replicate in mammalian cells without prior adaptation.

### Replication and pathogenicity of H3N3 virus in mice

**Figure 6:**
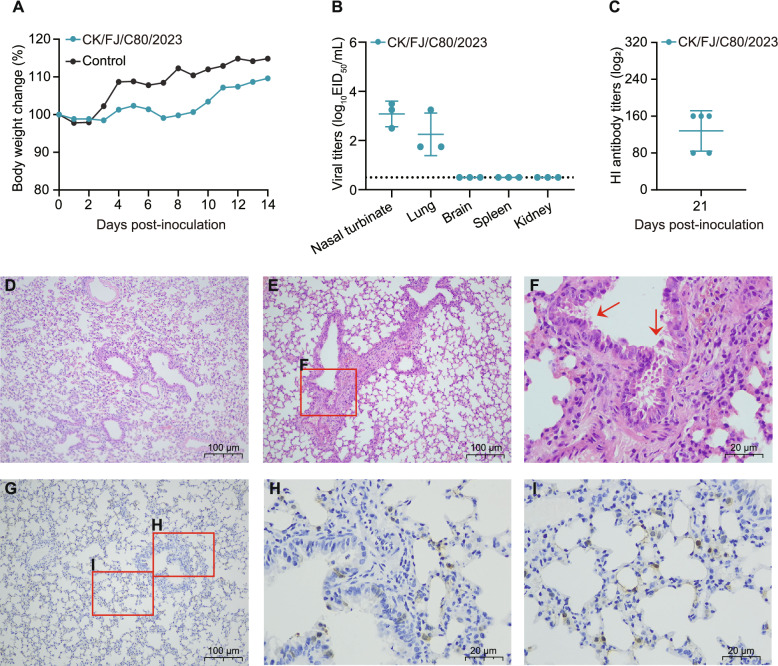
**Replication and virulence of H3N3 virus in BALB/c mice**. (**A**) Body weight changes of mice after inoculation with 10^6^ EID_50_ of the CK/FJ/C80/2023 virus. **B** Virus replication titres in indicated organs of mice after inoculation with 10^6^ EID_50_ of the CK/FJ/C80/2023 virus. The data shown represent the mean viral titres (*n* = 3) ± standard deviation. The dashed line indicates the lower limit of virus detection. **C** HI antibody titres in mice against the challenged H3N3 strain. Sera were collected from mice at 21 dpi. Haematoxylin and eosin (HE) staining of lungs of mice inoculated with (**D**) PBS or the H3N3 virus (**E**). The enlarged local version of the area is indicated by the red box in Figure **E** (**F**). Viral antigen of the H3N3 virus was detected in the epithelial cells (**G, H**, and **I**) of alveoli using immunohistochemical (IHC) staining. The enlarged local version of the area indicated by the red box in **G** is shown in **H** and **I**. Scale bars are represented in the figures.

To assess the pathogenicity of the H3N3 virus in mammals, we evaluated its replication and virulence in BALB/c mice. Briefly, eight BALB/c mice were intranasally inoculated with CK/FJ/C80/2023 virus at a dose of 10^6^ EID_50_. Five of these mice were monitored for weight loss for 14 dpi. The remaining three mice were euthanised on 3 dpi to collect nasal turbinates, lungs, brains, spleens, and kidneys for viral titration with embryonated chicken eggs. The results demonstrated that the H3N3 virus induced a modest reduction in body weight among mice inoculated with the CK/FJ/C80/2023 strain, as opposed to the PBS control group (Figure 6A).

Our findings showed that the H3N3 virus replicated in the nasal turbinates and lungs of the mice, with the virus titres ranging from 2.50 to 3.50 log_10_ EID_50_/mL and from 1.75 to 3.25 log_10_ EID_50_/mL, respectively. However, the virus was undetected in the brains, spleens, and kidneys (Figure 6B). The HI antibody titres on 21 dpi ranged from 80 to 160 (Figure 6C). Subsequently, we conducted sequencing of the PB2 gene from the virus recovered in the mice’s nasal turbinates and lung tissues, and no E627K or D701N mutations in the PB2 protein were detected.

The lungs were then prepared into pathological sections to verify the damage caused by the virus and to observe any pathological changes. The HE staining test revealed that the lungs of the control group mice exhibited normal structural integrity (Figure 6D). In the challenged group of mice, lung tissue exhibited damage and desquamation of epithelial cells in the bronchioles. However, the alveolar structure remained intact, with no evidence of inflammatory cell infiltration. Despite the absence of overt pathological alterations in the alveoli, as indicated by HE staining (Figures 6E and F), immunohistochemical (IHC) analysis revealed that the NP antigen was not only present within the bronchiolar epithelial cells but also exhibited positive distribution within the alveolar epithelial cells (Figures 6G–I).

These results demonstrated that the H3N3 virus could effectively replicate in the respiratory tract of mice without prior adaptation.

### Replication and transmission of H3N3 virus in chickens

**Figure 7:**
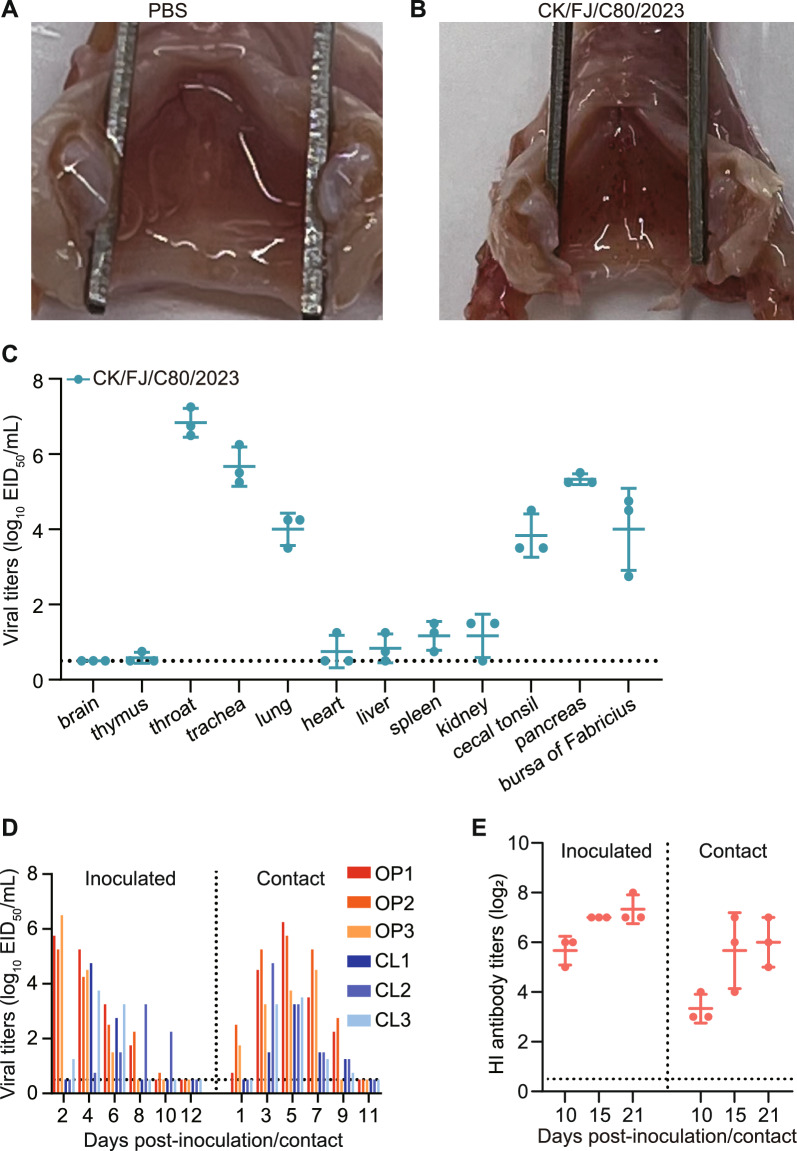
**Replication and transmission of the H3N3 viruses in chickens**. Tissue damage at the laryngeal inlet of chickens inoculated with **A** PBS or **B** H3N3 virus. **C** SPF chickens were inoculated with the CK/FJ/C80/2023 virus. The indicated organs were collected at day 3 dpi, and the viruses were titrated in embryonated chicken eggs. **D** Transmission of the H3N3 virus in chickens. Oropharyngeal and cloacal swabs were collected from the chickens at indicated times, and the virus was titrated in embryonated chicken eggs. **E** Serum from inoculated and contact chickens were collected at 10, 15, and 21 dpi to detect HI titres. The dashed lines indicate the lower limit of virus detection for panels C and D and the lower limit of HI antibody titres detection for **E**. OP: oropharyngeal swab; CL: cloacal swab.

We intranasally inoculated three chickens with the CK/FJ/C80/2023 virus with 10^6^ EID_50_ to investigate the virus’ replication dynamics and transmissibility. The chickens infected with the H3N3 virus showed signs of depression and diarrhoea, and within the first 3 dpi, a significant decrease in their food and water consumption was observed. Subsequently, the chickens showed a gradual recovery over the observation period. The birds were humanely euthanised at 3 dpi to assess viral titres across various organs. Compared with the control group, all three chickens showed haemorrhage at the laryngeal inlet (Figures 7A and B). Notably, viruses were detected in the larynx, trachea, lungs, cecal tonsil, pancreas, and bursa of Fabricius from all three chickens, with titres ranging from 2.75 to 7.25 log_10_ EID_50_/mL (Figure 7C). Viral titres were relatively lower in one or two chickens’ thymus, heart, liver, and kidney. However, viruses were detected in the spleens of three chickens, Figure 7C.

We then investigated the direct transmission ability of the CK/FJ/C80/2023 virus in chickens. Our findings showed that the virus was present in the oropharyngeal and cloacal samples collected from all three birds in the directly infected and contact groups during the observation period (Figure 7D). Seroconversion occurred in all the inoculated and direct contact birds. Furthermore, the virus triggered the production of HI antibodies in the inoculated and contact chickens with titres ranging from 5–8 log_2_ to 3–7 log_2_, respectively (Figure 7E).

These findings suggest that chickens are susceptible hosts of the H3N3 virus, which can replicate effectively in various organs and be transmitted efficiently in chickens via direct contact.

## Discussion

In recent decades, H3N3 influenza viruses have been detected in multiple animal species, including domestic poultry, pigs, seals, wild birds, and humans. Compared to the H3N2 and H3N8 AIVs, the H3N3 viruses exhibit a more restricted distribution, are less frequently detected, and have historically attracted less focus from researchers. Moreover, recently, a novel H3N3 strain emerged in China; however, its characteristics are mostly unknown.

This study identified a novel reassortant H3N3 virus in a live poultry market. We observed that this virus was pathogenic to chickens and could be transmitted through direct contact among chickens. Phylogenetic analysis has revealed that the H3N3 is a novel reassortant virus, characterised by the HA and NA genes derived from the H3N2 and H11N3 viruses, respectively. The internal genes of the H3N3 virus exhibit a complex evolutionary lineage originating from two distinct subtypes of AIVs. Specifically, the PB1, PA, and M genes exhibit a closer genetic affinity to those of H9N2 viruses, whereas the virus’ PB2, NP, and NS genes show genetic clustering with H3N8 viruses.

Previous studies have shown that the internal genes of these H3N8 viruses derive from H9N2 lineage genotype 57 [17, 18]. Further analysis revealed that PB2, NP, and NS genes of the H3N3 virus can be traced back to the H9N2 viruses [19]. Furthermore, according to the recently proposed classification of H9 subtype viruses [38], the H9N2 viruses, which donated their six internal genes to the H3N3 virus, belong to the B-lineage (BJ94, or Y280-like). This outcome indicates that the widely distributed H9N2 viruses are crucial for providing internal genes for the reassortment of novel AIVs in live poultry markets.

In contrast to the AIVs circulating in poultry farms that are mostly mitigated by extensive vaccination programmes, particularly for the H5 and H7 subtypes [9, 13, 39–42], live poultry markets in China remain significantly contaminated with AIVs. Therefore, birds without a strong pre-existing immunity will likely be infected with multiple sub-type AIVs when they stay in the live poultry markets [43].

While the presence of AIVs in live poultry markets does not necessarily indicate that identical viruses are also prevalent in poultry farms, the proximity between poultry and humans in these markets can lead to the potential transmission of AIVs to humans. Such an outcome was exemplified by the emergence of the H7N9 viruses in 2013 [1, 2, 44]. Therefore, it is imperative to develop and implement enhanced and more efficient strategies for the management of live poultry markets.

Previous research has categorised AIVs into three distinct groups based on the reduction in HA titre after treatment in a water bath at 56 °C [35, 37]. According to this criterion, the H3N3 virus exhibits relatively high levels of thermostability. Additionally, studies have shown that HA’s viral thermostability and acid stability are important determinants in AIV host range, infectivity, transmissibility, and human pandemic potentials [14, 35–37, 45–47]. The amino acid residues at positions 158D, 224N, 226Q, and 318I of HA in the H5N1 virus rendered the HA thermostability and contributed to viral transmission via respiratory droplets in ferrets [45]. The H5N1 virus’ HA activation pH exceeding 5.5 was conducive to replication and transmission within avian hosts. In contrast, an HA activation pH of 5.5 or lower was found to be advantageous for replication in the upper respiratory tract and for airborne transmissibility in ferrets [48, 49].

This distinction highlights the intricate relationship between HA activation pH and the viral ability to adapt to different hosts. Notably, the H3N3 virus does not show high acid stability compared to the human-adapted H1N1 virus [15]. However, the H3N3 virus does demonstrate notable thermostability. We speculate that this thermal stability may bolster the viral survival and transmission capacity, particularly in southeastern China’s warm and humid conditions. Nonetheless, further research is required to evaluate these potential risks comprehensively, especially as the virulence of AIVs is determined by multiple genetic factors [50].

This study also indicates that the newly emerged AIVs capable of replicating in mammals may acquire key amino acid mutations to adapt to new hosts, enhancing viral pathogenicity and facilitating human droplet transmission. The substitution of E627K or D701N in PB2 dramatically increased the virulence and transmissibility of the H7N9 viruses. Such an outcome may be attributed to PA protein as the intrinsic driving force behind the emergence of PB2 627 K during H7N9 replication in mammals [1, 14, 51]. Moreover, molecular analysis disclosed that the H3N3 virus in this study was absent of PB2 627 K or 701N mutations but harbouring 292 V and 588 V, which are known to enhance viral pathogenicity in mammals.

Here, multiple mammalian-adapted mutations in PB1, PA, NP, M1, M2, and NS1 were also detected, suggesting that the H3N3 virus may threaten mammals, potentially enhancing its capacity for transmission and pathogenicity within this host group. Interestingly, the H3N3 virus exhibited mild pathogenicity in mice, confining viral replication to the respiratory tract. Additionally, no PB2 627 K or 701N mutations were detected in viruses recovered from the lungs and nasal turbinates of mice at 3 dpi. Despite the H3N3 virus being categorised as a low pathogenic AIV subtype, the emerging novel H3N3 virus has shown a trend of increasing pathogenicity and efficient transmissibility in chickens. Although the H3N3 subtype virus in this study was replicated in mice without adaptation, our findings suggest that the virus is more adapted to chickens, indicating that effective control measures must be considered for poultry populations.

In summary, we identified a novel reassortant H3N3 virus, with internal genes derived from H3N8 and H9N2 viruses, in a live poultry market in China. Our research highlights the ongoing circulation and genetic reassortment of AIVs across various subtypes within domestic poultry populations. Thus, continued monitoring of AIVs in domestic and wild birds is imperative. It is particularly crucial to recognise their roles as reservoirs for the emergence of novel strains that may pose significant zoonotic risks to public health.

## Supplementary Information

**Additional file 1. The highest percentage of nucleotide identity of the A/chicken/Fujian/C80/2023 (H3N3) virus using BLAST method in GISAID and GenBank database.**

**Additional file 2. Molecular characteristics of the A/chicken/Fujian/C80/2023 (H3N3) virus.**

**Additional file 3. The potential glycosylation sites of HA and NA protein of the A/chicken/Fujian/C80/2023 (H3N3) virus.**

## Data Availability

The data supporting the conclusions of this article are included within the article. Additional data used and/or analysed during the current study are available from the corresponding author upon reasonable request. Supplementary material associated with this article can be found in the online version.
