# Supplementary material for: Emergence of a novel reassortant H3N3 avian influenza virus with enhanced pathogenicity and transmissibility in chickens in China
Source: Vet Res. 2025 Mar 11;56:56. doi: 10.1186/s13567-025-01484-1 (PMC11899391; doi:10.1186/s13567-025-01484-1)
Supplement: Supplementary file 1 — Additional file 1. The highest percentage of nucleotide identity of the A/chicken/Fujian/C80/2023 (H3N3) virus using BLAST method in GISAID and GenBank database. [file 13567_2025_1484_MOESM1_ESM.doc]

**Additional file 1 The highest percentage of nucleotide identity of the A/chicken/Fujian/C80/2023 (H3N3) virus using BLAST method in GISAID and GenBank database.**

| Gene | The highest similarity strain | Similarity of nucleotide | Accession number |
| --- | --- | --- | --- |
| HA | A/Environment/Guangdong/34268/2018 (H3N2) | 97.18% | EPI2210919 |
| NA | A/environment/Fujian/EV01/2020 (H11N3) | 98.16% | EPI2162127 |
| PB2 | A/chicken/Guangdong/F0210/2022 (H3N8) | 99.12% | EPI2047436 |
| PB1 | A/Guangdong/00470/2021 (H9N2) | 99.34% | EPI1884664 |
| PA | A/Guangdong/21ASF103/2021 (H9N2) | 99.40% | EPI2666662 |
| NP | A/Guangdong/ZS-23SF005/2023 (H3N8) | 99.60% | EPI2508607 |
| M | A/Guangdong/21ASF103/2021 (H9N2) | 99.49% | EPI2666661 |
| NS | A/chicken/Guangdong/14/2022 (H3N8) | 99.76% | EPI2594806 |
