# Supplementary material for: Emergence of a novel reassortant H3N3 avian influenza virus with enhanced pathogenicity and transmissibility in chickens in China
Source: Vet Res. 2025 Mar 11;56:56. doi: 10.1186/s13567-025-01484-1 (PMC11899391; doi:10.1186/s13567-025-01484-1)
Supplement: Supplementary file 2 — Additional file 2. Molecular characteristics of the A/chicken/Fujian/C80/2023 (H3N3) virus. [file 13567_2025_1484_MOESM2_ESM.doc]

**Additional file 2 Molecular characteristics of the A/chicken/Fujian/C80/2023 (H3N3) virus.**

| Protein | Amino acid mutations | Amino acid of CK/FJ/C80/2023 | Comments |
| --- | --- | --- | --- |
| HA | Cleavage site | PEKQTR/GLF | Highly pathogenic to chickens with multibasic HA cleavage site |
| HA1 | R62I | R | R→I: Increased the binding ability of the virus to human receptors |
|  | G78D | V | G→D: Reduced HA acid stability |
|  | H110Y | S | H→Y: Reduced the pH level necessary for viral fusion and enhanced thermal stability |
|  | A138S | A | A→S: Contributed virulence from influenza virus to mammalian hosts |
|  | A144G | V | A→G: Increased the binding ability of the virus to human receptors |
|  | N145S | N | N→S: Increased the binding ability of the virus to human receptors |
|  | I155T | T | I→T: Increased affinity for the human-type receptor |
|  | T160A | S | T→A: Increased transmission in guinea pigs |
|  | P162S | P | P→S: Enhanced pathogenicity to mice |
|  | L194P | L | L→P: Reduced acid stability and thermal stability |
|  | Q210R | Q | Q→R: Enhanced pathogenicity to mice |
|  | T212I | T | T→I: Reduced HA acid stability |
|  | G218E/W | G | G→E/W: Enhanced the pH level necessary for viral fusion |
|  | G219S | S | G→S: Reduced acid stability and thermal stability |
|  | Q226L | Q | Q→L: Increased virus binding to α 2,6-Sialic acid receptors |
|  | G228S | G | G→S: Increased virus binding to α 2,6-Sialic acid receptors |
| HA2 | I6M | I | I→M: Reduced acid stability and thermal stability |
|  | W47G | Q | W→G: Increased virulence in mice |
|  | K58I | K | K→I: Enhanced acid stability and thermal stability |
|  | G75R | G | G→R: Reduced acid stability and thermal stability |
|  | D112G | D | D→G: Enhanced the pH level necessary for viral fusion |
|  | T156N | T | T→N: Enhanced pathogenicity to mice |
| NA | K110E | E | K→E: Reduced susceptibility to zanamivir |
|  | V116A | V | V→A: Reduced susceptibility to zanamivir and Oseltamivir |
|  | I117T | T | I→T: Reduced susceptibility to oseltamivir and zanamivir |
|  | E119G | E | E→G: Resistant to neuraminidase inhibitors |
|  | Q136L | Q | O→L: Resistant to neuraminidase inhibitors |
|  | R152K | R | R→K: Resistant to neuraminidase inhibitors |
|  | H274Y | H | H→Y: Reduced susceptibility to oseltamivir and peramivir |
|  | R292K | R | R→K: Reduced susceptibility to Oseltamivir |
| PB2 | L89V | V | L→V: Enhanced polymerase activity, Increased virulence in mice |
|  | I292V | V | I→V: Enhanced replication efficiency and increased virulence in mice |
|  | A588V | V | A→V: Enhanced host adaptation |
|  | G590S | G | G→S: Enhanced replication efficiency |
|  | Q591K | Q | Q→K: Enhanced replication efficiency and increased virulence in mice |
|  | E627K | E | E→K: Enhanced polymerase activity and mammalian host adaptation |
|  | D701N | D | D→N: Mammalian host adaptation, increased virulence in mice |
| PB1 | H99Y | H | H→Y: H5 virus transmissible among ferrets |
|  | R207K | K | R→K: Decreased polymerase activity in mammalian cells |
|  | I368V | V | I→V: H5 virus transmissible among ferrets |
|  | H436Y | Y | H→Y: Decreased polymerase activity and virulence in mallards, ferrets and mice |
|  | M667T | I | M→T: Decreased virulence in mice |
| PA | K356R | R | K→R: Enhanced polymerase activity and viral replication |
|  | S409N | N | S→N: Enhanced the pathogenicity of the virus to mammals |
|  | T515A | T | T→A: Decreased polymerase activity in mammalian cells |
| NP | V286A | A | V→A: Increased virulence in mice |
|  | N319K | N | N→K: Enhanced replication efficiency |
|  | M437T | T | M→T: Enhanced replication efficiency |
| M1 | N30D | D | N→D: Increased virulence in mice |
|  | T215A | A | T→A: Increased virulence in mice |
| M2 | S31N | N | S→N: Reduced susceptibility to amantadine and Rimantadine |
| NS1 | P42S | S | P→S: Increased virulence |

Amino acids were analyzed by using MegAlign software.
