# Supplementary material for: Emergence of a novel reassortant H3N3 avian influenza virus with enhanced pathogenicity and transmissibility in chickens in China
Source: Vet Res. 2025 Mar 11;56:56. doi: 10.1186/s13567-025-01484-1 (PMC11899391; doi:10.1186/s13567-025-01484-1)
Supplement: Supplementary file 3 — Additional file 3. The potential glycosylation sites of HA and NA protein of the A/chicken/Fujian/C80/2023 (H3N3) virus. [file 13567_2025_1484_MOESM3_ESM.doc]

**Additional file 3** **The potential glycosylation sites of HA and NA protein of the A/chicken/Fujian/C80/2023 (H3N3) virus.**

| Protein | Potential glycosylation site | Amino acid |
| --- | --- | --- |
| HA | 24 | NST |
|  | 38 | NGT |
|  | 54 | NAT |
|  | 181 | NVT |
|  | 301 | NGS |
|  | 499 | NGT |
| NA | 57 | NCS |
|  | 66 | NNT |
|  | 72 | NIT |
|  | 146 | NGT |
|  | 308 | NET |

The potential glycosylation sites of HA and NA protein were predicted by using NetOGlyc-4.0.
